# Integrating Training on Professional Development in Graduate Public Health: Preliminary Insights From a Qualitative Study of MPH Alumni Perspectives

**DOI:** 10.1016/j.focus.2025.100397

**Published:** 2025-07-23

**Authors:** Yi Gao, Ariz Keshwani, Sumreen Akhtar, Leah C. Neubauer

**Affiliations:** 1 Department of Preventive Medicine, Northwestern University Feinberg School of Medicine, Chicago, Illinois; 2 University of Michigan School of Public Health, Ann Arbor, Michigan

**Keywords:** Professional development, public health training, experiential learning, graduate education, graduate public health, MPH students

## Abstract

**Introduction:**

For decades, researchers, educators, and policymakers have highlighted the benefits of well-designed course assignments in graduate settings, particularly those supporting learners in applying professional development objectives. Critically reflective course assignments enable learners to practice critical thinking and bridge gaps between theoretical knowledge and practical application. Despite its significant impact on overall competency and workforce outcomes, the integration and evaluation of experiential professional development training in academic public health curricula remain underexplored.

**Methods:**

This study examined the perspectives of 13 Master of Public Health global health alumni across 4 cohorts within a Council on Education for Public Health–accredited program. Participants provided insights into key learning outcomes, themes, and the learning impact of engaging in a threaded Professional Development Educational Event and Analysis course assignment. A pragmatic design-guided analysis approach was employed to identify recurring themes and insights.

**Results:**

Key learning outcomes included developing the habit of seeking and participating in professional development opportunities and incorporating topics beyond the formal Master of Public Health curriculum. Alumni emphasized the practical advantages of engaging in real-world settings and reflecting on their current and future public health practice.

**Conclusions:**

Findings suggest that incorporating innovative, active, and experiential learning opportunities, along with assignments fostering critical reflection, enhances the preparation of a competent and effective public health workforce. This approach bridges the gap between academic training and workforce readiness, offering actionable insights for curriculum development in public health education.

## Highlights

• Critical reflection enhances the impact of professional development assignments on career preparation.
• Engaging in real-world settings fosters practical skills and applied public health knowledge.
• Students value experiential learning opportunities that integrate networking, skill development, and career preparation.
• Alumni perspectives on career preparation can inform tailored professional development strategies in the Master of Public Health curriculum.

## INTRODUCTION

Professional development and career exploration opportunities within academic programs encompass a range of activities designed to cultivate practical knowledge, skills, and competencies that complement and extend theoretical knowledge.^1^ Engaging in professional development training is associated with higher levels of career readiness, satisfaction, retention, and other positive workforce outcomes.^1^, ^2^, ^3^ Experiential opportunities, such as networking events, informational interviewing, mentorship, and practice-based learning—including field placements, community-based projects, and internships—have increasingly become integral components of curricula across various disciplines.^1^^,^^4^

In recent decades, public health academic programs have placed a stronger emphasis on applied research and practice-based education within Master of Public Health (MPH) programs to better align with public health workforce needs.^5^^,^^6^ Competency frameworks, such as those established by the Council on Education for Public Health (CEPH),^7^ have contributed to significant adjustments in MPH program focus, design, curriculum, and pedagogical approaches.^6^^,^^8^^,^^9^ These shifts highlight the role of public health leadership development, workforce training, and academic–community partnerships for interprofessional experiential learning opportunities that aim to equip graduates with the necessary skills to address complex and evolving challenges in the public health field with confidence and competence.^4^, ^5^, ^6^, ^7^

Although the integration and evaluation of experiential professional development training in MPH curricula has been relatively underexplored,^4^ the documented benefits of appropriately designed assignments that put professional development objectives into practice across various disciplines underscore the need for further examination.^10^, ^11^, ^12^ Critically reflective course assignments have demonstrated learner benefits of bridging theoretical knowledge and real-world application, such as improved critical thinking and self-regulatory skills.^10^^,^^11^ Effective practices for professional development and formative guidance of future practice include active learning, peer interaction, practice and feedback, formative and summative assessments, and reflective thinking.^11^^,^^12^

One study on career and professional development training within a school of public health revealed that self-efficacy improved when students were encouraged to record insights and reflections from various career development activities, including learning workshops, community partnerships, and experiential opportunities. Implementing mandatory participation or cohort models in curriculum development, along with the early integration of community partnership opportunities, was identified as crucial for facilitating public health career exploration and professional development.^4^

There remains a pressing demand to identify individual perspectives on the skills valued and needed in the public health workforce and model promising practices in professional development within public health education and identify gaps in existing career preparation and professional development training programs.^6^^,^^13^^,^^14^ Assessing and prioritizing these learner needs can refine curricula, enhance professional development training in programs, and better prepare future public health practitioners to be drivers of public health systems change and population health improvement.^6^

In the broader context of expanding research on reflective practices in experiential public health professional development training opportunities and developing comprehensive approaches for meeting and prioritizing the professional development needs of MPH students, this exploratory study considers the insights and implications of a Professional Educational Event and Analysis assignment in an MPH global health course. The CEPH-accredited program was founded in 1996 in the Department of Preventive Medicine.

## METHODS

MPH alumni across 4 cohorts of the Global Health Concentration of Northwestern University’s Program in Public Health were surveyed through Qualtrics to gather perspectives on the concentration and the utility of curriculum components in a required three quarter, 30-week seminar course.

The survey included 5 demographic questions, and 10 open-ended, qualitative questions focused on the cohort model and 3 core seminar assignments: professional interview analysis, learning through failure, and professional educational event and analysis.

One question asked alumni to describe the most useful aspects of the Professional Development Educational Event and Analysis assignment, in which students develop professional development event learning objectives, incorporate instructor feedback, and attended a professional development event. Students then completed a critical analysis report that detailed the learning objectives in practice and reflected on the insights, learning outcomes, and impacts on their current and global health practice. Alumni across 4 cohorts (*n*=13) provided their views on the outcomes and impact of this assignment as part of the survey and received a $10 gift card for their participation.

Of 15 alumni contacted through email, 13 completed the survey. This study utilized reflexive thematic analysis, which involved an iterative process of coding and theme development to capture the learning experiences and perspectives of each student.^15^ Four members of the research team independently reviewed survey responses and manually developed a codebook to identify patterns and organize preliminary themes. The team met over multiple collaborative sessions to review and refine codes, assign final themes, and write an analytical narrative.

Analysis was guided by a pragmatic approach, which is rooted in identifying practical lessons and experiences.^16^ This approach allowed for the exploration of the aspects of the assignment that were perceived as most beneficial by the MPH alumni, with an emphasis on their practical implications for professional development.

Ethical approval for this research was obtained from the Northwestern University IRB on February 12th, 2022 (Number STU00215721).

## RESULTS

Findings from the critical pragmatically designed guided analysis identified several practical advantages of this assignment (Table 1). Recurring themes throughout responses found that alumni believed that the Professional Development Educational Event and Analysis assignment allowed for greater networking and career exploration opportunities as well as opportunities to learn or hone skills for their current and future public health practice.

**Table 1:** Describe the Most Useful Aspects of the Professional Development Event Assignment

Theme	Respondent	Quotes
Networking opportunities and expanding your network	1	Creating the connection with the colleague to work with them in the future and have them as someone I could get advice from.
	2	Meeting other individuals in public health and expanding my network.
	3	Again, it was a great opportunity to network with other public health professionals and opened the doors to future professional development opportunities. Northwestern has lots of seminars on various topics and it's so helpful to get involved early on.
	4	It was educational to hear different and often new perspectives on public health topics I was interested in. I think some of these events may have been able to be networking opportunities as well if approached correctly.
	5	Seeing and being able to speak to various organizations and professionals - expand network
New learning about topics	6	I learned a lot about different aspects of public health, and different niche professions people have in those areas.
	7	I learned about resources from the CDC that I had not heard of before.
	8	It was a purely educational session that informed me on the science of systems thinking.
	4	It was educational to hear different and often new perspectives on public health topics I was interested in. I think some of these events may have been able to be networking opportunities as well if approached correctly.
Engaging in professional development opportunities	9	It inspired me to think about what aspects of my job preparedness I wanted to improve upon or needed further guidance. It also helped me learn how to search for professional development opportunities.
	10	It gave me the habit of regularly checking interesting seminars near me that I could attend.
	3	Again, it was a great opportunity to network with other public health professionals and opened the doors to future professional development opportunities. Northwestern has lots of seminars on various topics and it's so helpful to get involved early on.
Honing and translating skills into practice	9	It inspired me to think about what aspects of my job preparedness I wanted to improve upon or needed further guidance. It also helped me learn how to search for professional development opportunities.
	11	The most useful aspects of the professional development assignment were the opportunities to hone different skills that I had not practiced previously. In particular, I was able to pull from my interest in writing/op-eds to find an event that involved global health and the utility of “translating” complex public health language into something that would be influential for several different fields.

### Networking Opportunities and Expanding Your Network

Several alumni described the benefits of speaking to various public health professionals during the professional development event, such as learning about resources or professions and making connections with other public health professionals. Two respondents noted the benefits of expanding [their] network through meeting various professionals or individuals in public health. One respondent highlighted the potential of connecting with a future colleague or being able to seek advice from them. In a similar vein, another respondent discussed how this “great opportunity to network with other public health professionals…opened the doors to future professional development opportunities.”

### New Learning About Topics

Alumni discussed how they gained new learning about public health topics as a result of this assignment. One respondent stated that “it was educational to hear different and often new perspectives on public health topics” that they were interested in. Another respondent similarly described “[learning] a lot about different aspects of public health and different niche professions,” highlighting how this assignment encouraged career exploration. Another respondent noted that they “learned about resources from the CDC that [they] had not heard of before.”

### Engaging in Professional Development Opportunities

Another recurring theme was learning how to establish the habit of seeking and engaging in professional development opportunities. Two respondents cited the benefits of attending seminars, with one noting that “it’s so helpful to get involved early on” and the other reflecting on how they developed “the habit of regularly checking interesting seminars near [them].” Another respondent noted that this assignment helped them “learn how to search for professional development opportunities.”

### Honing and Translating Skills Into Practice

The opportunity to hone practical skills was another important element of this assignment, and students reflected on how they considered aspects of their job preparedness that they “improve upon or needed further guidance” or “hone different skills that [they] had not practiced previously.” One respondent noted the value of learning and drawing from topics and skills outside of the formal MPH curriculum, such as their interest in writing op-eds and “the utility of ‘translating’ complex public health language into something that would be influential for several different fields.” This assignment encouraged students to identify strengths and areas of improvement, which allowed them to develop and hone diverse skills.

## DISCUSSION

The findings of this exploratory study indicate strong support for early involvement in experiential learning opportunities that enable MPH global health students in the program of public health to hone their professional development skills and bridge the gap between theoretical knowledge and practical application.

Alumni highlight the practical advantages of engaging in real-world settings and reflecting on their current and future public health practice through the Professional Development Educational Event and Analysis assignment. Individual alumni perspectives in this study identified specific components of professional development and career preparation that they prioritized. Their reflections demonstrated learning across multiple levels of Bloom’s Revised Taxonomy, from understanding and applying theoretical concepts in professional settings to analyzing and evaluating their experiences in relation to their ongoing professional development.^17^ Some synthesized their insights to create actionable strategies for pursuing further professional opportunities and shaping their career trajectories.

By participating and reflecting on professional development-oriented events, including seminars and workshops, the alumni appreciated gaining exposure to diverse public health topics and deepening their conceptual understanding of the field. In addition, they valued networking opportunities with professionals and organizations, speaking to the potential of these events to enhance career growth and development. Furthermore, the critical reflection aspect of the assignment played a pivotal role in honing metacognitive skills, allowing alumni to identify, organize, and evaluate areas in their ongoing professional development for improvement and growth.

Key outcomes and learning impacts include introducing them to career options; allowing them to build connections with global health professionals and reflect on personal goals; and encouraging them to develop the knowledge, habits, and skills needed to succeed in their current and future global health practice. These findings underscore the value of integrating opportunities for career exploration, professional development, and critical reflection within a course assignment.

### Limitations

Limitations of this study include the relatively small size of survey respondents (*n*=13) and the amount of time that passed for some alumni relative to their completion of the initial Professional Development Educational Event and Analysis assignment. Two respondents were unable to recall the specific aspects of this assignment and could not speak to its utility. Nevertheless, the response rate of the survey was 86.67% (13 of 15), implying a fairly strong willingness across 4 cohorts to reflect on their MPH global health education at Northwestern University and inform future curricular improvements throughout the program.

## CONCLUSIONS

Building a strong public health workforce and driving improvement in population health require responsive and ongoing professional development, training, and education. By embracing experiential learning and prioritizing professional development in graduate public health programs, academic institutions can support students in developing the knowledge, skills, and dispositions on the value of continuing professional education in public health. Findings from this study highlight the value of embedding opportunities for students to engage in active, experiential learning. Designing assignments that promote critical reflection and the application of complex concepts in real-world settings may help align MPH pedagogical models with dynamic public health workforce needs.

To prioritize the professional development needs of MPH students, academic institutions and public health programs should adopt comprehensive approaches that actively identify and integrate individual perspectives on career preparation and professional development training needs. This could entail regular monitoring and feedback mechanisms to evaluate students' expectations, interests, and preferences for future development opportunities. For CEPH-accredited units, criteria F1 Community Involvement in School or Program Evaluation & Assessment and B5 Alumni Perceptions of Curricular Effectiveness require the examination of usefulness and preparation on curriculum, coursework, and competencies.^8^ By incorporating these data, including student and alumni voices, in the curriculum design process, academic programs can tailor professional development initiatives to better serve the diverse needs of their learners. Ongoing faculty support in designing and implementing experiential learning activities is essential to sustaining the momentum of experiential learning. Further research is needed to assess the long-term impact of such assignments on students' career development and to investigate other opportunities for professional growth within the field of public health.
